# The Abuse of Astringents in the Treatment of Catarrh

**Published:** 1893-04

**Authors:** Charles M. Shields

**Affiliations:** Lecturer on Diseases of the Eye, Ear and Throat, Medical College of Virginia, Richmond


### THE ABUSE OF ASTRINGENTS IN THE TREATMENT OF CATARRH.*

By CHARLES M. SHIELDS, M. D.,
Lecturer on Diseases of the Eye, Ear and Throat, Medical College of Virginia, Richmond.

He referred more directly to acute nasal catarrh or "cold in the head," when we have feverishness, fulness and discomfort of the head, accompanied by watery mucous or muco-purulent discharges as more general characteristics, and argued that the idea of decreased discharges from nose in ordinary catarrh was rather paradoxical; that the original in Greek means to flow down, hence the general opinion that there is always an increased flow. This apparent increase in mucous secretions is not real, but the result of a diminution of the watery constituents of the

*Abstract of paper read at Richmond Academy of Medicine and Surgery February 28, 1893.

mucus, thus rendering it more viscid and tenacious, whereby its presence is more easily detected. That the lesson taught by Aschenbaudt and Keyser,that from twelve to fourteen ounces of fluid are necessarily absorbed by the air passing through the nose to bring it to the required degree of moisture saturation, showed clealy that the chief function of the nose is not olfaction but respiration. That the combination of turbinated-tissue, serum and muciparous secretions resulted in a consistency wherein the watery parts and solid parts are as ninety-three to seven, which under vaso-motor control adapted itself so nicely to the supply and demand of dry and moist atmospheric conditions that we were as a rule not conscious of its presence in a healthy nose. But when from some intrinsic or extrinsic cause the solid matter is increased at the expense of the fluid and serum exosmosis interfered with, what would have normally passed off by evaporation becomes more or less inspissated and acts as a local irritant to mucous membrane. This is the condition of affairs wherein the damaging effects of astringent sprays and powders are exhibited, which, if properly used in the very outset when lack of stimulation limited secretion and in the so-called purulent form more common among children, could do no special harm and might do some good ; while their more general use as powders and liquids hardened and excited the already hypertrophied glands, limited serum exosmosis and rendered the normal supply more tenacious. He thought that if his experience in this line had done him no other service, it had made clear to him that the ultimate result of continued use of astringents in the nasal cavity was harm rather than a benefit. In his paper he made exception in the use of caustics properly used in shrinking hypertrophic spots. He thought some of the most serious results of continued use of the drugs were mouth breathing, with all of its dire consequences, loss or prevented sense of smell, as olfaction could only be successfully accomplished when the parts were entirely normal as to size of naval cavity and condition of the mucous membrane. And perhaps the most frequent result was the hastening or actual causation of that *bête noir* of the rhinologist, atrophic catarrh, by rendering the parts abnormally dry. This seemed to him to be the result consequent upon the use of the so-called catarrh cures, put up by questionable doctors and druggists. He did not think it worth while to go far into the therapeutics of catarrh, as a recognition of the danger of the use of astringents, and strict adherence to cleanliness, such as weak alkaline antiseptic sprays and non-irritant snuffs, generally sufficed to put nasal cavity in normal condition. Of course proper caliber of cavity must be maintained by removal of foreign growths and correction of deflected septums.

